# Newborn with Refractory Seizures due to Hemimegalencephaly and Tuberous Sclerosis Complex: Case Report and Literature Review

**DOI:** 10.1055/a-2516-9103

**Published:** 2025-01-28

**Authors:** Mathies Rondagh, Linda S. de Vries, Lotte E. van der Meeren, Selma C. Tromp, Cacha M.P.C.D. Peeters-Scholte, Menno J.P. Toirkens, Sylke J. Steggerda

**Affiliations:** 1 Division of Neonatology, Department of Pediatrics, Willem-Alexander Children's Hospital, Leiden University Medical Center, Leiden, The Netherlands; 2 Department of Pathology, Leiden University Medical Center, Leiden, The Netherlands; 3 Department of Pathology, Erasmus Medical Centre, Rotterdam, The Netherlands; 4 Department of Neurology, Leiden University Medical Center, Leiden, The Netherlands; 5 Department of Radiology, Leiden University Medical Center, Leiden, The Netherlands

**Keywords:** hemimegalencephaly, neonatal seizures, tuberous sclerosis complex, postmortem, neuroimaging

## Abstract

Hemimegalencephaly (HME) is a rare congenital disorder that is initiated during embryonic development with abnormal growth of one hemisphere. Tuberous sclerosis complex (TSC), a genetic disorder, is rarely associated with HME.

We present a case of a newborn with HME with a confirmed mutation in the *TSC-1* gene and describe the clinical course, findings on amplitude-integrated electroencephalography (aEEG), cranial ultrasound (CUS), MRI, and the postmortem evaluation. Furthermore, we conducted a comprehensive literature review of all reported newborns with HME and a genetically confirmed TSC mutation.

This infant experienced therapy-resistant seizures after birth despite treatment with multiple antiseizure medications. CUS and MRI revealed HME of the left hemisphere. Early functional hemispherectomy, around the age of 3 months, was considered but dismissed after multidisciplinary evaluation, medical ethical consultation, and multiple discussions with the parents. Care was redirected due to worsening clinical and neurologic conditions, increasing respiratory insufficiency, and ongoing therapy-resistant seizures. Postmortem evaluation of the brain revealed hamartomatous brain changes and irregular gyration of the enlarged hemisphere. in addition, these changes were also present in the previously considered unaffected side, raising thoughts about the potential effectiveness of functional hemispherectomy.

This case report illustrates that in cases with TSC abnormalities might not be confined solely to the initially considered affected side. This can have important therapeutic implications.

## Background

Hemimegalencephaly (HME) is a rare congenital malformation of the brain, characterized by hamartomatous overgrowth of one hemisphere, with a prevalence of 1 to 3 cases out of 1,000 epileptic children. 1 These patients experience neurological manifestations such as (refractory) seizures, autism, developmental delay, hemianopia, and motor weakness. 1 Seizures in patients with HME are often treated with a wide range of antiseizure medications (ASMs), which are moderately effective. 2 The pathogenesis of HME is not fully understood. In most cases, HME is an isolated finding. Recently, it has been reported that somatic mutations of the *PI3K-AKT-mTOR* pathway might be associated with the development of HME. 3 The association between tuberous sclerosis complex (TSC), an autosomal dominant genetic disorder, and HME is rare and has been reported in a limited number of cases. 4 5 Approximately 85% of these cases are attributable to mutations in the *TSC1* or *TSC2* gene, with two-thirds of these mutations arising de novo. 6 TSC is responsible for encoding two distinct proteins, hamartin (TSC-1) and tuberin (TSC-2) which interact to form a heterodimeric complex. 4 This complex plays a critical role in the suppression of the mTOR signaling pathway. Alterations in one of these genes impair the regulation of cell growth and proliferation by the mTOR pathway, causing the development of tumors and lesions in various organs. The characteristic neuropathological features of TSC include subependymal nodules, cortical tubers, subependymal giant cell tumors, and various white matter abnormalities. 5 TSC also affects other organs and may lead to cardiac rhabdomyomas, renal angiomyolipomas, retinal nodular hamartomas, facial angiofibromas, and hypomelanotic macules. 2 We present a case of a newborn with HME based on a variant in the *TSC1* gene, assessed through continuous amplitude-integrated electroencephalography (aEEG), electroencephalography (EEG), cranial ultrasound (CUS), MRI, and postmortem evaluation. Furthermore, we provide a comprehensive review of the literature on newborns with HME and genetically confirmed TSC mutations and describe potential treatment options.

## Case Report

A female infant was born by secondary cesarean section due to non-progressive labor in a level 2 hospital at 38 weeks gestation. The pregnancy was uneventful, and no abnormalities were reported on prenatal ultrasound. Her birth weight was 3,740 g (+2 SD) with a length of 52 cm (+1 SD) and a head circumference of 34 cm (0 SD). Apgar scores were 7, 9, and 9 at 1, 5, and 10 minutes, respectively. No dysmorphic features or skin lesions were observed. The infant was supported with positive end-expiratory pressure without additional oxygen for 8 minutes, with recovery of spontaneous ventilation. Clinical seizures were observed 1 hour after birth, starting with grimacing followed by clonic jerking of arms and legs on both sides and sometimes the head. These episodes could not be interrupted. She was transferred to a level 3 hospital for neuromonitoring.

### Neurophysiology

**Fig. 1:**
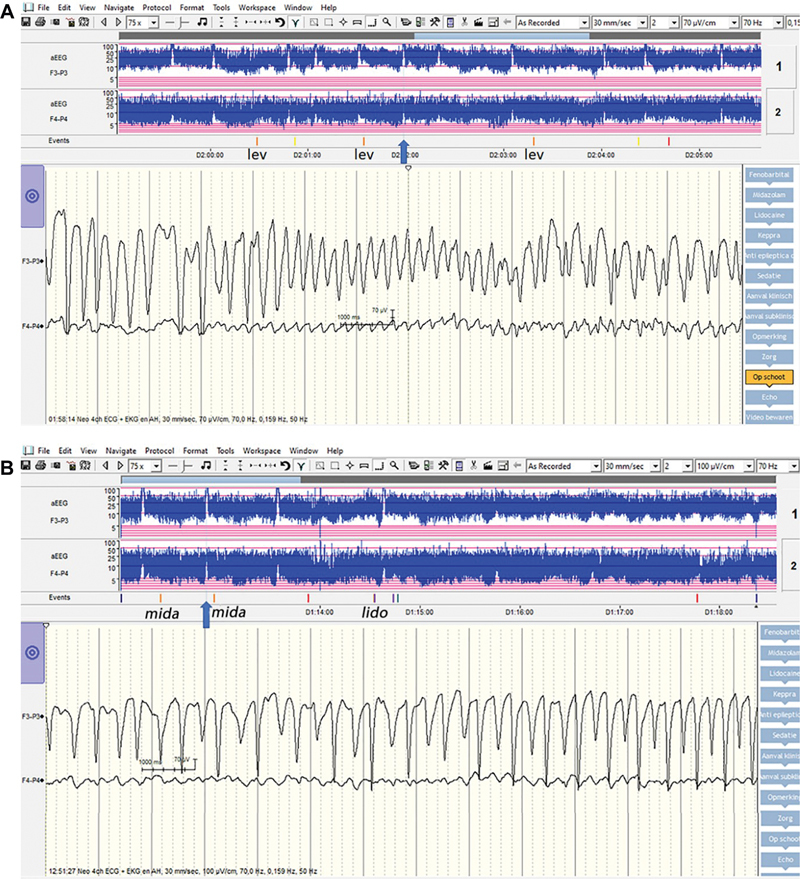
aEEG and EEG tracings of the infant with HME. The aEEG ( **A, B** ) of this infant showed a continuous, high-voltage pattern on the left hemisphere with a cut-off of the seizures. There was asymmetry in bandwidth between both hemispheres, the left is slightly narrower (F3-P3). Ictal discharges (arrow) were seen in both hemispheres. No seizure reduction was observed on aEEG after the administration of levetiracetam (lev) and midazolam (mida). Temporary effects of 4 hours and 20 minutes without epileptic activity were observed after the loading dose of lidocaine (lido). EEG ( **C, D** ) showed an interictal asymmetric background activity, with higher amplitudes and slower activity on the left hemisphere as compared with the right ( **C** ). The ictal activity consisted of high-voltage rhythmic discharges, most pronounced in the left occipital region, showing evolution in frequency and amplitude ( **D** ). aEEG, amplitude-integrated electroencephalography; EEG, electroencephalography; HME, hemimegaloencephaly.

The aEEG on arrival showed a continuous, high-voltage background pattern. There was an asymmetry in bandwidth between both hemispheres (left, F3-P3), narrower, and of higher voltage. Electrographic seizures, visible in both hemispheres, were high voltage on aEEG. The upper margin of the seizures in P3-F3 was cut off (at a standard scale of 0–100 μV). At the level 2 hospital, a cumulative dose of 40 mg/kg of phenobarbital was administered divided over three different doses. Nevertheless, seizures persisted. Due to persisting seizures, a continuous infusion of midazolam with a dose of up to 0.3 mg/kg/h was administered. Meanwhile, she developed respiratory insufficiency for which continuous positive airway pressure support was reinitiated. Due to increasing respiratory insufficiency, it was transitioned to non-invasive positive pressure ventilation. Due to refractory subclinical seizures on the aEEG, lidocaine was administered intravenously at a loading dose of 2 mg/kg over 10 minutes. Thereafter, a maintenance dose of 6 mg/kg/h was administered intravenously for 4 hours, 4 mg/kg/h for the next 12 hours, and eventually 2 mg/kg/h for the next 12 hours. Lidocaine had a temporary effect, with seizure freedom lasting for 4.5 hours but afterward, epileptiform activity was observed once more on the aEEG ( Fig. 1A, 1B ). Repetitive seizure activity was seen with approximately two seizures per hour, despite the administration of the aforementioned ASMs. Therefore, additionally, levetiracetam was administered up to a loading dose of 60 mg/kg, without any effect. Both the high-voltage brain activity of the left hemisphere and the seizures were confirmed on a full 19-channel EEG ( Fig. 1C, 1D ). This showed an asymmetrical background pattern, with normal patterns according to age on the right hemisphere and higher amplitudes with slower activity on the left hemisphere. Occasionally, rhythmical discharges of very high amplitude were observed mainly in the left occipital region with evolution in frequency and amplitude, corresponding with the seizures observed on the aEEG.

**Figure:**
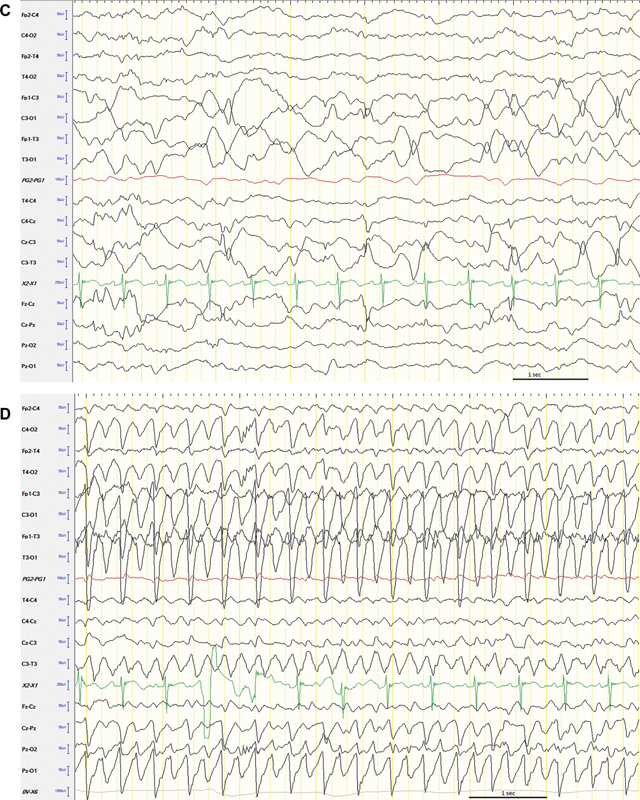


### Neuroimaging

**Fig. 2:**
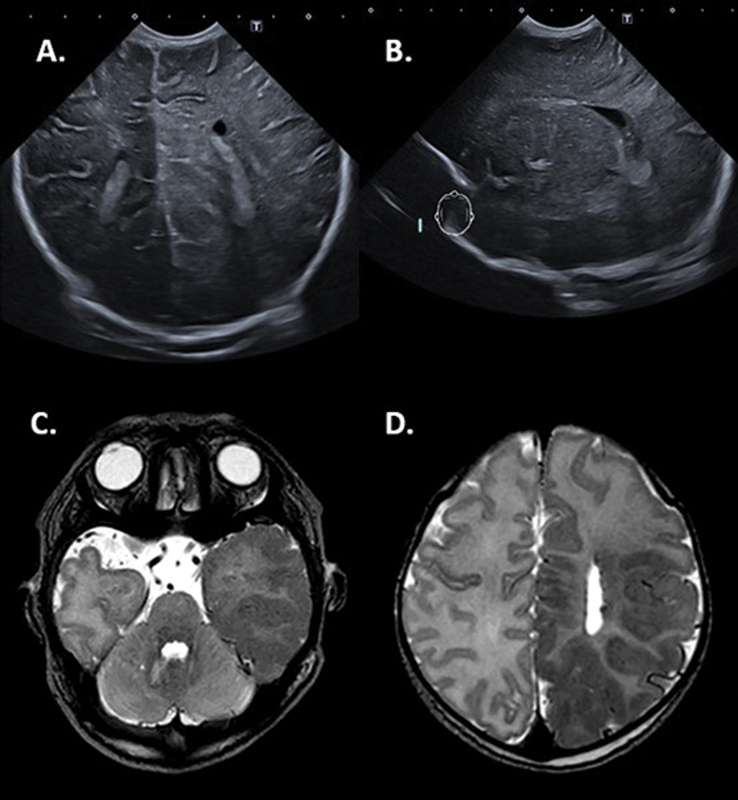
CUS and MRI images of the brain illustrating HME. Cranial ultrasound ( **A, B** ) showed an enlarged left hemisphere and cortical blurring of the left parieto-occipital structures. Increased echo density in the white matter on both the coronal and sagittal views was seen. T2-weighted MRI images showing a deviant hypointense thickened left temporal ( **C** ), and parieto-occipital cortex ( **D** ) (pachygyria) with decreased gyration in the left hemisphere. Subependymal noduli were present on the left side. CUS, cranial ultrasound; HME, hemimegaloencephaly.

Postnatal CUS showed an asymmetric, enlarged left hemisphere with increased echogenicity, loss of normal cortical demarcation, and an abnormal gyration pattern ( Fig. 2A, 2B ), all suggestive of HME. Cortical blurring was particularly evident in the left parieto-occipital region of the brain. A 3-T MRI confirmed the diagnosis of HME, showing the asymmetric enlarged left hemisphere with a hypointense aspect (on T2-weighted images) of the left parietal, occipital, and temporal lobe with thickening of the cortical ribbon ( Fig. 2C, 2D ) (pachygyria). Also, subependymal nodules were present in the left hemisphere. No abnormalities were noted in the right hemisphere.

### Treatment Options and Ethical Considerations

A multidisciplinary medical ethical consultation involving specialists from pediatric neurology, neurosurgery, genetics, perinatology, neonatology, and ethics discussed treatment options. Functional hemispherectomy was considered in consultation with the national referral center for epilepsy surgery in the Netherlands, but at that time hemispherectomy was not performed before 3 months, due to the high risk of complications at this young age. In the case of a functional hemispherectomy, this would involve bridging refractory epilepsy for 3 months at the neonatal intensive care unit (NICU) with therapy-resistant seizures and possibly ongoing mechanical ventilation. The predicted highest achievable outcomes, as described by the referral center, were the possibility of walking, limited use of the right hand, cognitive impairments with special education, and reduced communication ability. The parents expressed concern about the projected future and their child's quality of life. Meanwhile, respiratory insufficiency worsened despite a significant reduction in midazolam levels and the infant's clinical and neurologic condition deteriorated. In light of the therapy-resistant seizures, worsening respiratory insufficiency, and expected long-term neurodevelopmental impairments, the decision was made to redirect care. Thereafter, the infant died at the hospital in the presence of the parents and permission for postmortem was given.

### Postmortem Examination

Macroscopic HME with a larger left hemisphere and a firmer appearance was confirmed ( Supplementary Fig. S1 [available in the online version only]). Irregular gyration was seen in both hemispheres. Notably, the irregular gyration and hamartomatous changes in the previously considered unaffected right hemisphere were not detected by earlier MRI. The cerebellum appeared macroscopically normal and the fourth ventricle was not enlarged. The meninges had a normal aspect. Histology showed an abnormal lamination in the left enlarged hemisphere compared with the right hemisphere with an increase in rhabdoid cells with spherical eosinophilic cell bodies and large round–oval eccentrically located nuclei with a prominent nucleolus. These changes were accompanied by a variably extensive gemistocytic response. In addition, a subependymal giant cell astrocytoma (2 mm) was present. No histological abnormalities were observed in the cerebellum. In addition to the brain, the heart exhibited TSC characteristics, with six cardiac rhabdomyomas varying from 0.5 to 5 mm in diameter. Whole exome sequencing in plasma revealed a somatic pathogenic nonsense variant in the *TSC1* gene ( *TSC1* , *Chr9* ; *GRCh37* ) confirming the diagnosis of TSC. The mutation was not identified in the parents (non-related Caucasians) indicating de novo occurrence.

## Discussion

We present a case of a female infant born at 38 weeks, who developed refractory seizures early after birth, based on HME. She was assessed using aEEG, EEG, CUS, MRI, and genetic evaluation. Because of the severe clinical condition, the inability to achieve seizure control despite multiple ASMs associated with increasing respiratory insufficiency, and a poor neurodevelopmental prognosis it was decided to redirect intensive care. Postmortem revealed hamartomatous brain changes and irregular gyration was seen in both hemispheres and a de novo pathogenic mutation in the *TSC1* gene was found. Bilateral brain involvement could well have had a negative effect on the success of functional hemispherectomy.

Although several case reports have documented newborns with HME and TSC, a comprehensive review of the literature on newborns with HME and genetically confirmed TSC has not been performed. HME with genetically confirmed TSC was reported in five other cases in the literature ( Table 1 ). 2 5 6 7 8 Four out of five neonates had mutations in the *TSC1* gene. 2 5 6 8 All neonates developed seizures in the first days after birth. The efficacy of ASMs was found to be limited in one of the reported cases, who subsequently was given the mTOR inhibitor everolimus; however, no mention was made of the effect. 5 Shim et al and Guerra et al, described the MRI diagnosis of brain abnormalities in the normal-appearing hemisphere, including cortical tubers, band heterotopia, subependymal hamartoma, and white matter lesions. 2 7 In these two reported cases, functional hemispherectomy was performed at, respectively, 5.5 weeks and 27 months of age. Shim et al reported a favorable outcome, achieving seizure freedom by 12 months of age. 2 In contrast, Guerra et al reported an unfavorable outcome with no improvement following hemispherectomy. 7 The effectiveness of functional hemispherectomy in neonates with contralateral abnormalities due to TSC remains therefore unclear. Serletis et al reported an infant who exhibited significant developmental delay. 5 Cuddapah et al and Tinkle et al described favorable long-term outcomes, including seizure freedom for several years following hemispherectomy. 6 8 Both Cuddapah et al and Shim et al showed that functional hemispherectomy in patients with HME and TSC is feasible below the age of 3 months. 2 6

**Table 1:** Neonatal cases of hemimegaloencephaly with genetically confirmed tuberous sclerosis complex in the literature

Study (year of publication)	Rondagh et al (2024)	Shim et al (2022) 2	Serletis et al (2022) 5	Cuddapah et al (2015) 6	Guerra et al (2007) 7	Tinkle et al (2005) 8
Gender	Female	Female	Female	Male	Male	Female
Gestational age and birth weight	38 weeks, 3,740 g	37 weeks, 3,200 g	37 weeks, 2,760 g	Full-term, 3,500 g	Unknown, 3,620 g	Both unknown
Genetic testing	TSC1 (Chr9;GRCh37)	TSC-1 mutation (c.17431744insCAAGG)	TSC-1 mutation (intron 16)	TSC-1 mutation (nucleotide 2074)	TSC-2 mutation (exon 37)	TSC-1 mutation
HME	Left	Left	Left	Left	Left	Left
Clinical presentation	Starting with grimacing followed by clonic jerking of arms and legs on both sides and sometimes the head	Macrocephaly Respiratory insufficiency due to a weak cry and reduced activity. Seizures starting on day 4 after birth	Micrognathia, right facial droop, left eye anterior dysgenesis, ptosis, and enlarged fontanelles. Respiratory insufficiency. The right hemi-body had decreased movements and increased tone, with frequent jerking in the right arm/leg	Macrocephalic. No dysmorphic features. Seizures at home	Macrocrany, cranial asymmetry, bilateral microphthalmia, and severe partial tonic seizures	Seizures and hypothyroidism
TSC features (without postmortem)	Subependymal nodules	Multiple cardiac rhabdomyomas, renal cysts, and several tubers in the right cerebral hemisphere	Two truncal ash leaf spots, a hypomelanotic macule, and cardiac rhabdomyoma	Three hypopigmented macules on the trunk	Multiple cardiac rhabdomyomas, and two shagreen patches	Several abnormally bright foci in the subcortical white matter of both hemispheres
Used ASM and efficacy	Phenobarbital, midazolam, levetiracetam and lidocaine; no effect	Phenobarbital, topiramate, vigabatrin; no effect	Midazolam, phenobarbital, levetiracetam, topiramate and vigabatrin; limited effect	Phenobarbital, levetiracetam; no effect	Phenobarbital, midazolam, phenytoin; no effect	Not reported
mTor inhibitor	Not used	Not used	Everolimus (5 mg/kg/day), the effect not described	Not used	Not used	Not used
Surgical intervention	No surgical intervention	Functional hemispherectomy (at 5.5 weeks of age)	Anatomical hemispherectomy (at 6.5 weeks of age)	Functional hemispherectomy (at 7 weeks of age)	Functional hemispherectomy (at 27 months of age)	Anatomical hemispherectomy (at 5 months of age)
(fetal) Ultrasound	No fetal ultrasound	Fetal ultrasound showed ventriculomegaly of the left lateral ventricle. Neonatal ultrasound also showed band heterotopia of the right hemisphere	Not described	Not described	Not described	Not described
MRI	Asymmetric enlarged left hemisphere with a hypointense aspect (on T2-weighted images) of the left parietal, occipital, and temporal lobe with thickening of the cortical ribbon. Also, subependymal nodules were present in the left hemisphere. No abnormalities were noted in the right hemisphere	Several tubers and band heterotopia in the right hemisphere and HME in the left hemisphere	The left hemisphere is thickened in keeping with diffuse pachygyria, associated with blurred irregularities along the gray-white interface. In comparison, the right cerebral hemisphere and brainstem appear relatively unaffected	Unilateral enlargement of the left parietal and occipital lobe demonstrating broad and thick gyral pattern with diminished sulcation, consistent with pachygyria. There is an asymmetrical enlargement of the left occipital horn with a somewhat parallel rather than converging configuration.	In the left hemisphere a diffuse hamartomatous enlargement with ventriculomegaly, displacement of the midline structures and cerebellar tentorium, thick cortex with lissencephaly in the posterior region, and agyric-pachygyric appearance in the frontal lobe, broad gyri with shallow sulci, blurring of the corticomedullary junction. Hypoagenesis of the corpus callosum was observed. The right hemisphere (unaffected HME side) showed a subependymal hamartoma in the temporal horn of the lateral ventricle and focal linear areas of hyperintensities in the frontal white matter consistent with dysmyelination	Enlarged left hemisphere with abnormal cortical thickening, polymicrogyria, and heterotopic gray matter
EEG	Asymmetrical background pattern, with normal patterns on the right hemisphere according to age, and higher amplitudes with slower activity on the left hemisphere. Occasionally, rhythmical discharges of very high amplitude were observed mainly in the left occipital region with evolution in frequency and amplitude	Subclinical seizures with evolving ictal rhythmic discharges from the left occipital (T3-O1) or centrotemporal areas (Fp1-T3, T3-C3). The subclinical seizures spread to the contralateral side	Serial EEGs showed left centrotemporal seizures recurring every few seconds, lasting 2.5–10 minutes	Frequent left hemispheric rhythmic spike and wave discharges, maximum in the central parietal and temporal regions, lasting 2–4 minutes, and interrupted by delta/theta slowing of less than 1-minute duration. Both the electroencephalographic findings and depressed mental status were most consistent with non-convulsive status epilepticus	Repetitive EEG discharges of sharps and sharp waves on the left side, involving almost the whole hemisphere, during awake and sleep, alternating to generalized depressed activity; the right side showed discontinuous activity during quiet sleep and brief periods of continuous activity during active sleep, with abnormal sharp transients in the frontal areas. The frequent left EEG discharges, often lasted more than 60 seconds	Not performed
Histology	Abnormal lamination in the left enlarged hemisphere compared to the right hemisphere with an increase in rhabdoid cells with spherical eosinophilic cell bodies and large round-oval eccentrically located nuclei with a prominent nucleolus. These changes were accompanied by a variably extensive gemistocytic response	Not performed	Histology revealed abnormalities affecting the entire excised hemisphere, rather than focal clusters typical of tubers. Medium-sized balloon cells were clustered in both gray and white matter, expressing immunohistochemical features of astroglial lineage	Disorganized cortical architecture lacking normal lamination, maloriented cortical neurons, white matter with microcalcification and gemistocytic astrocytosis, and balloon cell were observed in tissue after hemispherectomy	Not performed	Not performed
Neurological outcome	Redirection of care	Clinical seizures recurred in the form of infantile spasms at 4 months of age. Postoperative EEG performed at 5 months of age revealed subclinical seizures originating from the right hemisphere. At the age of 12 months, clinical seizures did not recur	At 3 years from surgery, severely delayed, she has made slow progress, now rolling and holding her head unsupported	Remained seizure-free nearly 5 years after functional hemispherotomy surgery. At 2.5 years, mild right hemiparesis persists, Wechsler Preschool and Primary Scale of Intelligence, third edition, scores included a Full-Scale intelligence quotient of 91 (27th percentile), Verbal Index Score of 93 (32nd percentile), and Performance Index Score of 90 (25th percentile). Measures of receptive vocabulary, and visuospatial and constructional ability were in the normal range.	Intractable epilepsy at the age of 27 months, she underwent a functional hemispherotomy without improvement	At 3.5 years of age, she had remained seizure-free for approximately 2 years after anatomical hemispherorotomy and was making significant developmental progress

Abbreviations: ASM, antiseizure medication; EEG, electroencephalography; HME, hemimegaloencephaly; TSC, tuberous sclerosis complex.

Seizures in newborns with HME are typically difficult to control with medication. 2 Functional hemispherectomy is often the only treatment to provide effective seizure control. 1 Recently, one case was described that underwent an anatomical hemispherectomy at 6.5 weeks of age for refractory seizures in HME due to *TSC1* gene. Afterward, the infant remained seizure-free for at least 1 year. 5 In our case, HME was associated with a mutation in the *TSC1* gene, and postmortem observation revealed extensive brain abnormalities in the enlarged left hemisphere, thereby confirming the findings on MRI. However, in addition, it also revealed hamartomatous brain changes and alteration in gyration on the previously considered unaffected right side, not detected by MRI. These abnormalities on the previously considered unaffected right side likely play a crucial role in determining the success of epilepsy surgery. This highlights the importance of gaining a thorough understanding of abnormalities within the brain (tubers, subependymal nodules, subependymal giant cell astrocytomas) in patients with HME based on TSC mutations before considering a functional hemispherectomy. 9 PET with 2-deoxy-2-(18F)fluoro-d-glucose (FDG) and ictal Single Photon Emission Computed Tomography have been identified as valuable tools for localizing (non- and epileptogenic) tubers and could provide additional localization data to standard modalities in pre-epilepsy surgical evaluation. 9 The aEEG pattern showed an asymmetric background pattern with ictal discharges and continuous high voltage activity in the most severely affected hemisphere. Including this case report, only four cases of HME with corresponding aEEGs tracings have been reported in the literature. 10 11 12 Three out of four newborns with HME showed the characteristic continuous high-voltage pattern on aEEG in one hemisphere, with high-voltage ictal discharges, that appeared to be cut off at the top on a regular scale, which is not described in other diseases. 10 11 12 When this characteristic and asymmetric aEEG pattern is observed in newborns with seizures, it is suggestive of HME, even before conducting CUS or MRI.

Our case report highlights the challenge of managing therapy-resistant seizures and predominantly subclinical seizures in infants with HME, as monitored with aEEG and EEG. Only a temporary effect of lidocaine, with reduced seizure activity, was observed. Lidocaine can be used as an ASM for acute provoked seizures but can only be administered for a short period (36–48 hours) as prolonged use will lead to the accumulation of methylethylglycinexylidide (MEG), which can result in seizures and cardiac side effects. 13 The administration of mTOR inhibitors emerged as a potentially beneficial treatment. Recent studies have shown promising results for mTOR inhibitors in preliminary clinical studies of patients affected by TSC, for both seizure reduction and other disease-modifying effects. 14 Recently, a neonate with HME without TSC started treatment with rapamycin, an mTOR inhibitor, due to intractable epilepsy pending hemispherectomy. 15 Within a week, seizure frequency was reduced by >50%, and developmental improvements were observed. Surgery was delayed and ultimately performed when the patient was 5.5 months old. This highlights the potential of mTOR inhibitors as a bridging therapy for infants awaiting surgical intervention. In our case, no mTOR inhibitors were used, as at the time the results of the preliminary clinical studies of mTOR inhibitors were not yet available. Based on recent studies, currently, mTOR inhibitors should be considered in patients with TSC, as they significantly reduce seizure frequency and have a tolerable safety profile. 14

## Conclusion

This case illustrates that early detection of HME through CUS/MRI and characteristic aEEG/EEG patterns is crucial, alongside consideration of underlying genetic conditions like TSC. It is important to be aware that the contralateral hemisphere may also be affected in TSC, despite normal MRI findings, which can impact the overall outcome. These insights underscore the importance of a comprehensive, genetics-informed approach in managing HME.
