# Supplementary material for: Newborn with Refractory Seizures due to Hemimegalencephaly and Tuberous Sclerosis Complex: Case Report and Literature Review
Source: Neuropediatrics. 2025 Jan 28;56(2):133–41. doi: 10.1055/a-2516-9103 (PMC11932765; doi:10.1055/a-2516-9103)
Supplement: Supplementary file 1 — Supplementary Material [file 10-1055-a-2516-9103-s0920243878sc.pdf]

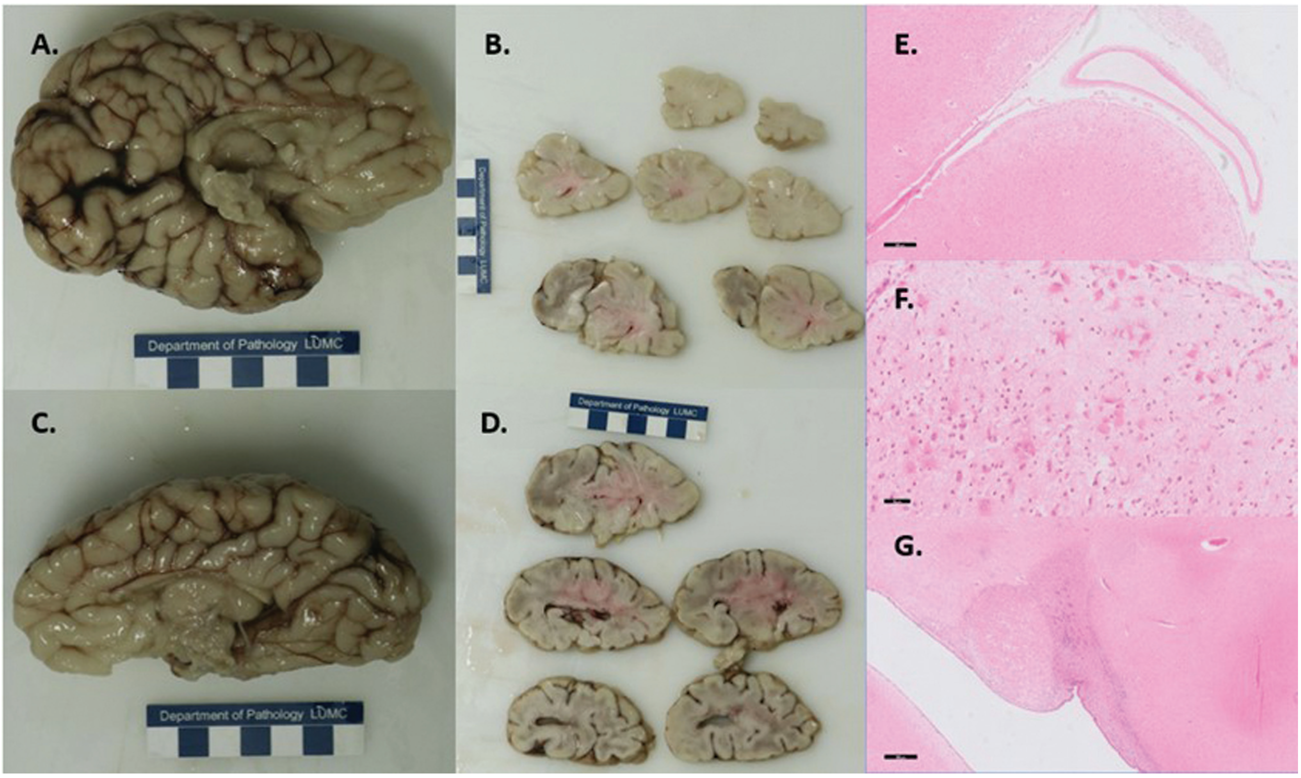

Supplementary Fig. S1 Postmortem. Hemimegaloencephaly with enlarged, firmer left hemisphere (**A, B**) and irregular gyration in both hemispheres (**A–D**). Abnormal lamination in the left hemisphere (**E**, HE 20 $\times$) with increased rhabdoid cells (**F**, HE 200 $\times$), extensive gemistocytic response, and a 2-mm subependymal giant cell astrocytoma (**G**, HE 20 $\times$).

 © 2025. The Author(s).
